# The SCFA Receptor GPR43 and Energy Metabolism

**DOI:** 10.3389/fendo.2014.00085

**Published:** 2014-06-05

**Authors:** Ikuo Kimura, Daisuke Inoue, Kanako Hirano, Gozoh Tsujimoto

**Affiliations:** ^1^Department of Applied Biological Science, Graduate School of Agriculture, Tokyo University of Agriculture and Technology, Tokyo, Japan; ^2^Department of Pharmacogenomics, Kyoto University Graduate School of Pharmaceutical Science, Kyoto, Japan

**Keywords:** GPR43, FFAR2, SCFA, gut microbiota, energy metabolism

## Abstract

Free fatty acids (FFAs) are essential nutrients and act as signaling molecules in various cellular processes via binding with FFA receptors. Of these receptors, GPR43 is activated by short-chain fatty acids (SCFAs; e.g., acetate, propionate, and butyrate). During feeding, SCFAs are produced by microbial fermentation of dietary fiber in the gut, and these SCFAs become important energy sources for the host. The gut microbiota affects nutrient acquisition and energy regulation of the host and can influence the development of obesity, insulin resistance, and diabetes. Recently, GPR43 has been reported to regulate host energy homeostasis in the gastrointestinal tract and adipose tissues. Hence, GPR43 is also thought to be a potential drug target for metabolic disorders, such as obesity and diabetes. In this review, we summarize the identification, structure, and activities of GPR43, with a focus on host energy regulation, and present an essential overview of our current understanding of its physiological roles in host energy regulation that is mediated by gut microbiota. We also discuss the potential for GPR43 as a therapeutic target.

## Introduction

Obesity is currently one of the most serious public health problems worldwide because of its increasing prevalence and contribution to serious metabolic disorders, including type-2 diabetes (1, 2). Obesity is the result of a long-term imbalance between energy intake and expenditure, and is therefore regulated by multiple pathways involving metabolites, hormones, and neuropeptides (3). Excess food intake, especially high-fat and sugar foods, and lack of physical activity are considered as risk factors in the developing of obesity. Recent research has demonstrated that the gut microbiota is involved in obesity and metabolic disorders (4, 5). An important role of the gut microbiota is to catabolize substrates, such as dietary fiber, that are not completely hydrolyzed by host enzymes during host feeding (6). The main colonic bacterial fermentation products of dietary fiber are short-chain fatty acids (SCFAs), such as acetate, propionate, and butyrate (7). SCFAs can be used for *de novo* synthesis of lipids and glucose, which are the main energy sources for the host (8).

## The SCFA Receptor GPR43

In addition to functioning as an energy source, SCFAs are also essential nutrients that act as signaling molecules. Recently, two orphan G-protein coupled receptors (GPCR), GPR41 and GPR43, were reported to be activated by SCFAs. During ligand screening for bioactive compounds, researchers reported that GPR43, also known as free fatty acid receptor 2 (FFAR2), was activated by acetate using Ca^2+^ assays in transfected cells (9, 10). GPR43 can also be activated by other SCFAs, including propionate and butyrate; acetate and propionate are the most efficient for activating GPR43, followed by butyrate and then other SCFAs (9, 11).

GPR43 is a dual-coupling GPCR that binds with the pertussis toxin-sensitive G_i/o_ and G_q_ proteins (11). Stimulation of GPR43 by SCFAs inhibits cAMP production, activates the extracellular signal-regulated kinase (ERK) cascade via interactions with the G_i/o_ family of G-proteins, increases intracellular Ca^2+^ levels, and promotes activation of the mitogen-activated protein kinase (MAPK) cascade via interactions with the G_q_ family of G-proteins. However, the physiological significance of this GPR43-based dual-coupled signaling mechanism is still unclear. GPR43 is expressed in the adipose tissue, intestines, and immune tissues (12, 13). In the immune system, many studies have investigated the role of GPR43 in regulating inflammatory responses (13–15). These results indicate that GPR43 is important for gut immunity involving gut microbiota and food. On the other hands, GPR43 expression in adipose and gastrointestinal tissues suggests that GPR43 may be involved in energy regulation (16); moreover, reverse transcription polymerase chain reaction (RT-PCR) in mouse tissues has shown that *Gpr43* is expressed in white adipose tissue (WAT) and the intestine (12).

## Adipose Tissues

Adipose tissues are very important tissues associated with energy homeostasis and energy accumulation. In adipose tissues, GPR43 may be involved in regulating obesity and energy accumulation, Similarly, *Gpr43* mRNA is expressed in WATs, including subcutaneous, perirenal, and epididymal tissues, as well as in 3T3-L1-derived adipocytes and mature adipocytes (12). Based on the observed expression of *Gpr43* in adipose tissues and adipocytes, Hong et al. performed a series of studies to elucidate the functions of GPR43 in adipocytes (12). They showed that *Gpr43* expression was significantly greater in the WAT of mice with high-fat diet (HFD)-induced obesity compared with normal chow-fed mice. Moreover, in 3T3-L1 cells, treatment with SCFAs, increased *Gpr43* and *Pparg* transcript levels, while suppression of *Gpr43* mRNA by RNA interference inhibited adipogenesis. Thus, SCFAs appear to promote adipogenesis via GPR43. Additionally, in 3T3-L1 derived adipocytes, SCFAs suppress isoproterenol-induced lipolysis in a concentration-dependent manner (12). Ge et al. demonstrated that these effects are dependent on GPR43 using Gpr43-deficient mice (17). That is, they showed that acetate suppressed lipolysis, and release of glycerol occurred in a concentration-dependent manner in adipocytes isolated from wild-type mice *in vitro*, and the activation of GPR43 by intraperitoneal injection of sodium acetate instantly reduced plasma fatty acid *in vivo*; these effects were abrogated in *Gpr43*-knockout mice (17). In brown adipose tissues (BATs), which have a central role in the regulation of energy balance and homeostasis, Bjursell et al. reported that *Gpr43*-knockout mice fed an HFD exhibited improved insulin sensitivity in old age due to increased energy expenditure, which resulted in increased body temperature (18). As a potential explanation for this, histological observation of BAT in *Gpr43*-knockout mice revealed that these mice exhibited decreased lipid dispersion compared with wild-type mice fed an HFD. However, we could not detect *Gpr43* expression in BATs (19). Hence, further studies are needed to elucidate the role of GPR43 in energy control via BAT.

Recent evidence suggests that the gut microbiota affects host nutrient acquisition and energy regulation and is therefore related to obesity, insulin resistance, and diabetes in the host (20–22). During feeding, SCFAs, which act as ligands for GPR43, are produced by microbial fermentation of dietary fiber in the gut. Hence, we examined the relationship between gut microbiota and systemic energy regulation by GPR43 in adipose tissue using *Gpr43*-mutantand germ-free mice (19). In a series of *in vitro* and *in vivo* studies, we found that *Gpr43* deficiency induced obesity in mice, while mice that overexpress *Gpr43* only in adipose tissues were lean under normal conditions; both of these mouse strains did not exhibit either phenotype under germ-free conditions or after antibiotic treatment. Furthermore, SCFA-mediated GPR43 activation suppressed adipose insulin signaling, leading to inhibition of fat accumulation in the adipose tissue, and unincorporated lipids and glucose were primarily utilized in muscles. That is, the expression of energy expenditure-, glycolysis-, and beta-oxidation-related genes increased, while the expression of gluconeogenesis-related genes decreased in the muscles of *aP2-Gpr43* TG mice. However, the mechanism by which GPR43 mediated the suppression of insulin signaling in adipocytes is not mediated by cAMP inhibition, but instead involves the beta and gamma subunits of the G_i/o_ protein, not G_q_ protein. Thus, GPR43 acts as a sensor for excessive dietary energy, thereby controlling body energy utilization while maintaining metabolic homeostasis. The GPR43-insulin pathway in adipose tissue may function as an important physiological mechanism through which these metabolic fuels regulate body energy balance. Hence, these previous reports in adipose tissues indicate that GPR43 has potential therapeutic relevance for the treatment of metabolic disorders, such as obesity and type-2 diabetes.

## Intestinal Tissues

In the intestines, GPR43 may be involved in regulating appetite and insulin signaling. Indeed, *Gpr43* mRNA has been shown to be expressed in rat and human ileum and colon, especially in enteroendocrine cells (23, 24). Like adipose tissue, the intestine is also critical for energy homeostasis, as supported by its association with secretion of appetite gut hormones and nutrients absorption (25, 26). Using immunohistochemistry analysis with GPR43 antibodies in rats, Karaki et al. reported that GPR43 is expressed in peptide YY (PYY)-containing enteroendocrine L-cells of the gastrointestinal tract (23). Enteroendocrine L-cells are also one of the major cell types that express the proglucagon genes *GLP-1* and *GLP-2*. GLP-1 and GLP-2 proteins are co-stored and co-secreted with PYY from enteroendocrine L-cells (27), and SCFAs are co-secreted with GLP-1 from mixed colonic cultures via GPR43 *in vitro* and *in vivo* (28). Quantitative RT-PCR (qRT-PCR) showed that *Gpr43* and *Gpr41* were abundantly expressed in GLP-1-secreting L-cells. Moreover, SCFAs raised cytosolic Ca^2+^ through G_q_ signaling pathways in L-cells in primary culture. *Gpr43*- or *Gpr41*-knockout mice exhibited reduced SCFA-mediated GLP-1 secretion both *in vitro* and *in vivo* and have impaired glucose tolerance. Additionally, *Gpr43*-knockout mice exhibited reduction of insulin secretion in accompaniment with the reduction of *in vivo* glucose-stimulated GLP-1 secretion (28). However, to determine the effects of SCFAs on the secretion of gut hormones, the expression and function of GPR41, and GPR43 in subtypes of enteroendocrine cells, such as L-cells and K-cells, must be characterized in detail using *Gpr41*- and *Gpr43*-double-knockout mice. Thus, pharmacological manipulation of appetite using a GPR43 agonist may be useful for treatment of obesity. Moreover, these types of studies may provide essential information concerning the role of GLP-1 in insulin secretion in patients with type-2 diabetes. The anorexigenic neural circuits are subsequently activated via PYY and GLP-1, reducing food intake and increasing energy expenditure. Hence, regulation of PYY and GLP-1 secretion via GPR43 maintains energy homeostasis and may be a valid approach for treating metabolic disorders.

## Conclusion

**Figure 1:**
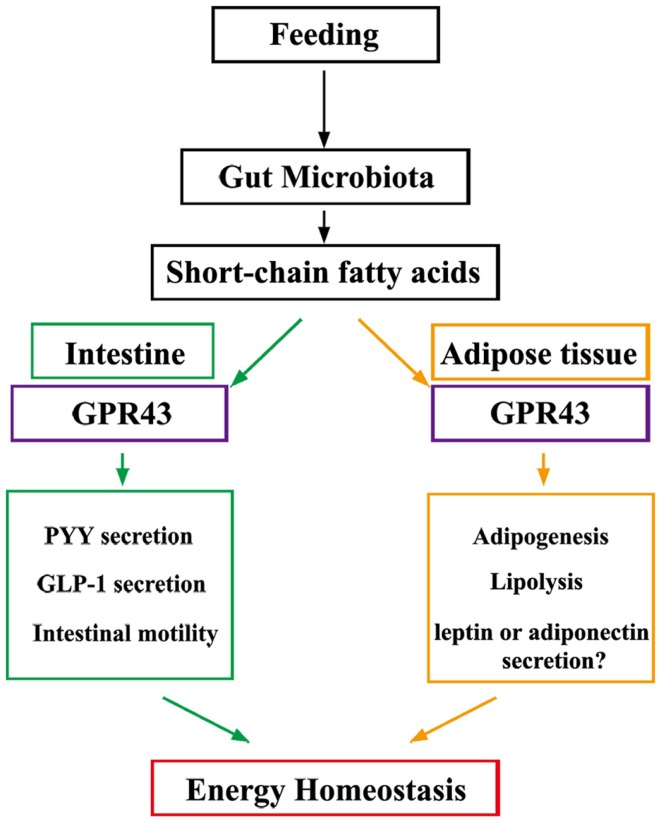
**Under “fed” conditions, SCFAs are produced in the gut by bacterial fermentation of dietary fiber**. SCFAs alter the metabolic rate by stimulating GPR43 in adipocytes and promote gut hormone secretion and motility by stimulating GPR43 in the intestine, thereby increasing energy expenditure and improving glucose tolerance to increase energy utilization.

GPR43 regulates metabolic rate when activated by SCFAs that are produced by gut microbiota in a variety of host tissues (Figure 1). Future studies are expected to reveal the presence of a central mechanism that mediates the effects of diet and probiotics on human homeostasis. Additionally, GPR43 may represent a promising therapeutic target for the treatment of metabolic syndromes, such as obesity and diabetes.

## Conflict of Interest Statement

The authors declare that the research was conducted in the absence of any commercial or financial relationships that could be construed as a potential conflict of interest.
